# Lower cerebral blood flow is associated with faster cognitive decline in Alzheimer’s disease

**DOI:** 10.1007/s00330-016-4450-z

**Published:** 2016-06-22

**Authors:** Marije R. Benedictus, Annebet E. Leeuwis, Maja A.A. Binnewijzend, Joost P.A. Kuijer, Philip Scheltens, Frederik Barkhof, Wiesje M. van der Flier, Niels D. Prins

**Affiliations:** 1 Alzheimer Centre & Department of Neurology, Neuroscience Campus Amsterdam, VU University Medical Centre, PO Box 7057, 1007 MB Amsterdam, The Netherlands; 2 Department of Physics and Medical Technology, Neuroscience Campus Amsterdam, VU University Medical Centre, Amsterdam, The Netherlands; 3 Department of Radiology and Nuclear Medicine, Neuroscience Campus Amsterdam, VU University Medical Centre, Amsterdam, The Netherlands; 4 Department of Epidemiology & Biostatistics, Neuroscience Campus Amsterdam, VU University Medical Centre, Amsterdam, The Netherlands

**Keywords:** perfusion, prognosis, progression, dementia, Alzheimer’s disease

## Abstract

**Objective:**

To determine whether lower cerebral blood flow (CBF) is associated with faster cognitive decline in patients with Alzheimer’s disease (AD).

**Methods:**

We included 88 patients with dementia due to AD from the Amsterdam Dementia Cohort. Mean follow-up was 2 ± 1 years. Linear mixed models were used to determine associations of lower whole brain and regional pseudo-continuous arterial spin labelling measured CBF with rate of cognitive decline as measured with repeated mini-mental state examination (MMSE). Model 1 was adjusted for age, sex, and education. Model 2 was additionally adjusted for normalized gray matter volume, medial temporal lobe atrophy, white matter hyperintensities, microbleeds, and lacunes. Analyses were repeated after partial volume correction (PVC) of CBF. Statistical significance was set at *p* ≤ 0.05.

**Results:**

Patients were 65 ± 7 years old, 44 (50 %) were women, and mean baseline MMSE was 22 ± 4. Annual decline (β[SE]) on the MMSE was estimated at -2.11 (0.25) points per year. Lower whole brain (β[SE]-0.50[0.25]; *p* ≤ 0.05) and parietal (β[SE]-0.59[0.25]; *p* < 0.05) CBF were associated with faster cognitive decline. PVC cortical CBF was not associated with cognitive decline.

**Conclusions:**

Lower CBF, in particular in the posterior brain regions, may have value as a prognostic marker for rate of cognitive decline in AD.

***Key points*:**

*• In AD, lower CBF is associated with more rapid cognitive decline.*

*• Decreasing CBF does not reach a plateau early in AD.*

*• PcASL-CFB has additive value to conventional structural MRI measures in AD.*

## Introduction

Alzheimer’s disease (AD) is a progressive neurodegenerative disorder and one of the aspects that determines progression is cognitive decline. In AD, cognitive decline appears to be largely variable between individual patients [1, 2] and predictors of cognitive decline in patients with dementia due to AD are currently largely lacking [3]. Moreover, with the introduction of new research criteria for preclinical AD [4], the focus of research is shifting more and more towards prognostic factors in the early, or preclinical, phase of AD [5]. Factors that predict decline in early phases of the disease, however, may lack prognostic value once patients are diagnosed with dementia [6].

Cerebral blood flow (CBF) may be a relevant prognostic factor for the rate of cognitive decline in patients with AD. CBF can be measured with arterial spin labelling (ASL) and is found to be lower in AD patients compared to controls [7–9]. Decreased CBF is thought to reflect synaptic failure [10–12]. Synaptic dysfunction continues throughout the course of AD [13] and is still associated with cognitive decline in later stages of AD [14]. Lower ASL-CBF has been found to predict conversion from mild cognitive impairment (MCI) to AD [15]. Moreover, a lower ASL-CBF has been associated with worse cognition, even in the stage of AD dementia [8]. Using single-photon emission computed tomography (SPECT), fast declining AD patients also appeared to have a lower baseline CBF than more slowly declining patients [16, 17].

Several previous reports show that ASL-CBF is able to predict progression in healthy controls and MCI patients [15, 18]. At present it is unknown whether ASL-CBF also has prognostic value for the rate of disease progression in patients with dementia due to AD. We aimed to investigate whether CBF measured with ASL is associated with the rate of cognitive decline in patients with AD.

## Methods

### Patients

**Fig. 1:**
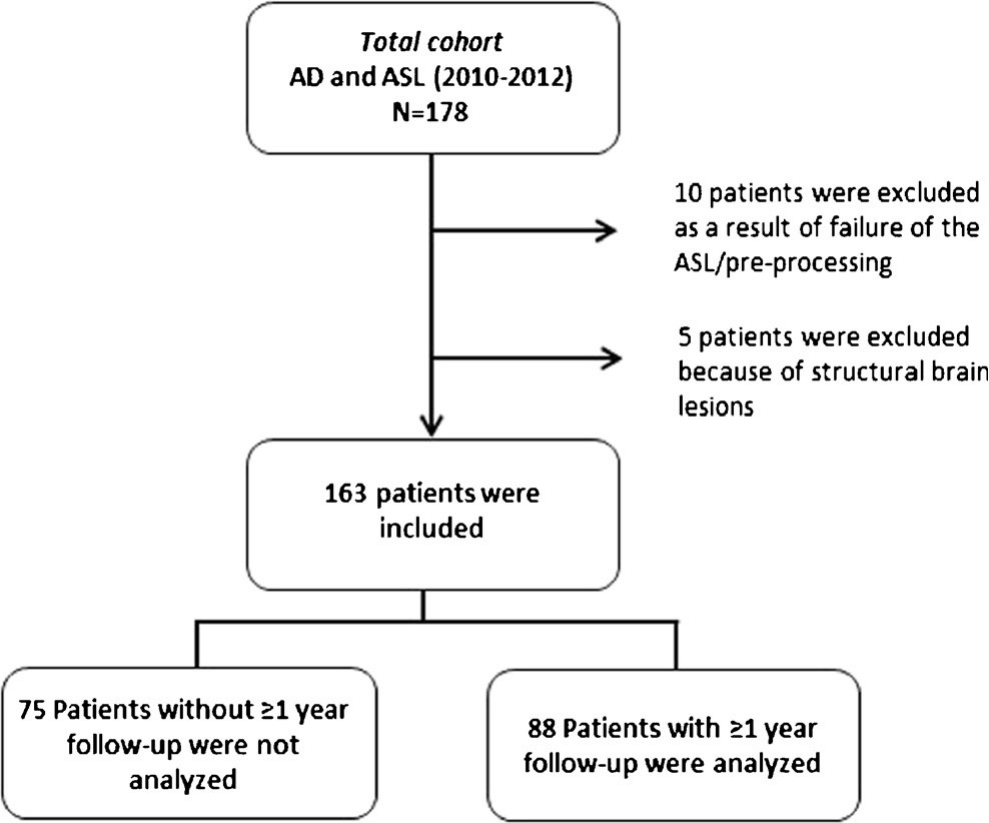
Flow chart of patient inclusion

From the Amsterdam Dementia Cohort [19], we selected all AD patients who underwent a pseudo-continuous ASL (pcASL) MRI scan during 2010-2012 (n = 178, Fig. 1). We excluded patients with structural brain lesions (n = 5: two with post-traumatic lesions; two with brain tumour; one with a large recent haemorrhage) and patients for whom pre-processing of the ASL MRI data failed (n = 10); this resulted in a potential dataset of 163 AD patients with available ASL. Of these, 88 patients met our inclusion criterion of at least two MMSE scores available over at least 1 year of follow-up. Excluded patients had on average a lower MMSE score (19 ± 5 vs. 22 ± 4, *p* < 0.01), but both groups were comparable with regard to demographics and MRI characteristics (data not shown).

Standardized work-up included a physical and neurologic examination, extensive neuropsychological testing, laboratory tests, and brain magnetic resonance imaging (MRI). All results were discussed in a multidisciplinary meeting, after which the diagnosis “probable AD” was made according to the NINCDS-ADRDA criteria [20], and all patients fulfilled the core clinical criteria of the NIA-AA [21] (more details on this standardized work-up can be found in van der Flier et al [19]). For all patients we had information about education, classified using the Verhage scale [22].

The medical ethics committee of the VU University Medical Center approved the study. All patients provided written informed consent to use their clinical data for research purposes.

### MRI acquisition

MRI scans were performed on a 3 T whole body MR system (SignaHDxt, GE Medical Systems Milwaukee, WI, USA) using an 8-channel head coil. The scan protocol included T1-weighted, T2-weighted, fluid-attenuated inversion recovery (FLAIR), and gradient echo T2*-weighted images. Medial temporal lobe atrophy (MTA) was rated on the oblique reconstructions of the T1-weighted images, using a 5-point rating scale (0-4) [23]. For analyses we used the mean of left and right MTA scores. White matter hyperintensities (WMH) were assessed using the Fazekas scale on the FLAIR images, with scores from 0 to 3 [24]. Microbleeds were defined as small round foci of hypointense signal, up to 10 mm in brain parenchyma on T2*-weighted images. Lacunes were defined as deep lesions (3-15 mm) with CSF-like signal on all sequences. Microbleeds and lacunes were counted. The rater was blinded to the patients’ clinical data.

PcASL [25, 26] perfusion images (3D-FSE acquisition with background suppression, post-label delay 2.0 s, echo time = 9 ms, repetition time = 4.8 s, spiral readout 8 arms x 512 samples; 36 × 5.0 mm axial slices, 3.2 × 3.2 mm in-plane resolution, reconstructed pixel size 1.7 × 1.7 mm, acquisition time 4 min) were calculated using a single compartment model [27] after the subtraction of labeled from control images. CBF was described by using the following equation:

$$ CBF=\lambda\;\left(1-{e}^{-{T}_{SAT}/{T}_{1GM}}\right)\kern0.5em \frac{e^{w/{T}_{1B}}}{2\kern0.5em {T}_{1B}\left(1-{e}^{-\tau /{T}_{1B}}\right)\;\varepsilon}\frac{\varDelta S}{S_0} $$

with post-label delay w = 2.0 s, labelling time τ = 1.5 s, partition coefficient λ = 0.9, labelling efficiency ε = 0.8*0.75 (label PCASL * background suppression), T1 of blood T_1B_ = 1.4 s, SR time for PD image T_SAT_ = 2.0 s, and correction for SR in PD image T_1GM_ = 1.2 s. ΔS stands for ASL difference image and S_0_ for proton density-weighted reference image.

### Post-processing of MRI data

Both T1-weighted and pcASL images were corrected for gradient non-linearities in all three directions. Further data analyses were carried out using FSL (version 4.1; http://www.fmrib.ox.ac.uk/fsl). Processing of T1 images consisted of non-brain tissue removal [28], linear registration to standard space [29] and tissue segmentation [30] yielding partial volume estimates. These steps are conveniently combined in FSL Sienax, which additionally produces a normalized gray matter volume (NGMV) with reference to the MNI standard brain. CBF maps [31] were linearly registered to the brain-extracted T1 images. Partial volume estimates were transformed to the ASL data space and used in a regression algorithm [32], using a 3D Gaussian kernel of 9.5 mm full width at half maximum, to create a partial volume corrected (PVC) cortical CBF map. Partial volume estimates were subsequently used as a weighting factor to calculate corrected cortical CBF. The MNI152 atlas and the Harvard-Oxford cortical atlas (both part of FSL) were used to create regions-of-interest (ROIs) of the frontal, parietal, precuneus, and posterior cingulate cortex (PRCPCC), temporal and occipital brain areas, to extract mean uncorrected and PVC CBF values for each region. Additionally, the uncorrected mean whole brain CBF was calculated as the average perfusion of all voxels classified as brain tissue, including CSF.

### Cognitive follow-up

Follow-up took place by clinical routine visits to our memory clinic. All patients had at least one follow-up, no less than 1 year after baseline. At follow-up the MMSE was used as a measure of general cognitive function [33].

### Data analysis

We used SPPS version 20 (for Windows) for the statistical analyses. Linear mixed models were applied to assess the associations for whole brain and regional CBF (frontal, parietal, PRCPCC, temporal, and occipital) with rate of decline on the MMSE. A linear mixed model has increased statistical power as it accounts for within-person correlations over time, allows different numbers of assessments and accounts for varying time intervals between assessments. CBF was analysed per standard deviation decrease (SD), as a step of 1 mL/100 g/min may be too small to be informative. All MMSE assessments, including those at baseline were taken into account. A random intercept and random slope with time (in years) were assumed, meaning that the model accounted for individual variation of change in MMSE over time. The model included terms for the CBF measurement, time, the interaction between CBF and time (all as independent factors), and covariates. The dependent variable consisted of all MMSE scores. Model 1 was adjusted for age, sex, and education. In model 2, we additionally adjusted for NGMV, MTA, WMH, microbleeds, and lacunes. Next, we repeated these analyses (without adjustment for NGMV in model 2) using PVC cortical CBF. Statistical significance was set at *p* < 0.05.

## Results

Table 1 presents the demographics, MRI characteristics, and CBF measurements of the patients in the study. Patients had a mean age of 65 ± 7 years and 44 (50 %) were women. Baseline MMSE was 22 ± 4, and average follow-up was 2 ± 1 years. The total number of MMSE tests that were included in the analyses was 277. Median number of MMSE tests within one patient was three, with a minimum of two and a maximum of eight. Annual change (β[SE]) in MMSE was estimated at -2.11(0.25) points per year.

**Table 1:** Patient demographics

	n = 88
Age (years)	65 ± 7
Female sex ^a^	44 (50 %)
Follow-up time (years)	2 ± 1
Level of education (Verhage scale)	5 ± 1
Baseline MMSE score	22 ± 4
Median number of MMSEs^b^	3 (2 - 8)
Annual change in MMSE ^c^	-2.11 ± 0.25
MRI characteristics	
Normalized gray matter volume (ml)	715.8 ± 44.6
Medial temporal lobe atrophy^b, d^	1.5 (0-3)
White matter hyperintensities^b, e^	1 (0-3)
Microbleeds^b^	0 (0-100)
Lacunes^b^	0 (0-2)
Cerebral blood flow (mL/100 g/min)	
Whole brain	28.0 ± 5.6
PVC cortical	43.0 ± 8.7
Regional cerebral blood flow (mL/100 g/min)	
Frontal	18.7 ± 4.7
Parietal	23.9 ± 5.9
PRCPCC	30.2 ± 6.8
Temporal	21.8 ± 5.1
Occipital	29.1 ± 7.7
Regional PVC cerebral blood flow (mL/100 g/min)	
Frontal	43.2 ± 10.0
Parietal	47.3 ± 10.9
PRCPCC	53.8 ± 11.8
Temporal	39.0 ± 8.3
Occipital	48.7 ± 10.7

Availability for incomplete data: Level of education 87/88; Microbleeds 86/88

Data are represented as mean ± standard deviation, patients with variable present (%)^a^ or median (range) ^b^.

^c^ Calculated with linear mixed models, to make use of all available MMSE values. Given value is the unadjusted main effect of time.

Key: *MMSE*, mini-mental state examination; *PVC*, partial volume corrected.

^d^ Medial temporal lobe atrophy was rated with a visual rating scale (0-4).

^e^ White matter hyperintensities were rated with the Fazekas scale (0-3).

As expected, whole brain CBF was lower than PVC cortical CBF, as the former also contains cerebrospinal fluid and white matter.

Table 2 shows results of the linear mixed models we used to investigate the associations between baseline CBF with baseline MMSE and with annual change in MMSE. CBF measures were not associated with MMSE at baseline. Adjusted for age, sex, and education (model 1), lower whole brain CBF was associated with faster decline on the MMSE (β[SE]: -0.50[0.25], *p* = 0.05). When looking at region specific CBF, we found that in particular lower parietal CBF was associated with a more rapid cognitive decline. Lower occipital CBF tended to be associated with more rapid cognitive decline, although this association did not reach significance (*p* = 0.06). CBF in the other regions was not associated with cognitive decline. When we performed additional adjustments for structural MRI measures (model 2), the associations remained largely comparable.

**Table 2:** Cerebral blood flow and cognitive decline

	Model 1		Model 2	
	Estimated Baseline MMSE	Estimated annual change in MMSE	Estimated Baseline MMSE	Estimated annual change in MMSE
Whole brain CBF ^a^	-0.42 ± 0.38	-0.50 ± 0.25*	-0.10 ± 0.40	-0.50 ± 0.25*
*Regional CBF* ^a^				
Frontal	-0.16 ± 0.41	-0.13 ± 0.26	0.29 ± 0.43	-0.13 ± 0.26
Parietal	-0.63 ± 0.38	-0.59 ± 0.25**	-0.22 ± 0.44	-0.59 ± 0.25**
PRCPCC	-0.60 ± 0.38.	-0.41 ± 0.25	-0.17 ± 0.42	-0.43 ± 0.25
Temporal	-0.31 ± 0.37	-0.46 ± 0.25	-0.10 ± 0.41	-0.45 ± 0.25
Occipital	-0.39 ± 0.37	-0.47 ± 0.25^¥^	-0.05 ± 0.40	-0.46 ± 0.25^¥^

Data are represented as β ± SE. Linear mixed models were used to investigate associations between CBF and change in MMSE. A random intercept and random slope for time (in years) were assumed. The model includes terms for the CBF measure, time, the interaction between the CBF measure and time and covariates. The βs for estimated baseline MMSE represent the estimated additional change in z-score associated with a standard deviation decrease in CBF at baseline. The βs for estimated annual change in MMSE represent estimated additional change in z-score for each year of follow-up.

Abbreviations: *MMSE*, mini-mental state examination; *CBF*, cerebral blood flow.

Model 1: adjusted for age, sex, and education.

Model 2: additional adjustment for normalized gray matter volume, medial temporal lobe atrophy, white matter hyperintensities, microbleeds, and lacunes.

** *p* = 0.02

* *p* = 0.05

^¥^ *p* = 0.06

^a^ CBF was inverted (i.e. higher is worse) and given per standard deviation increase (worsening). Negative βs indicate that a worse CBF is associated with a decline in MMSE.

Whole brain PVC cortical CBF was not associated with annual decline on the MMSE (model 1: β[SE] -0.39 [0.25], n.s.). In addition, we found no associations between regional PVC cortical CBF and annual decline on the MMSE (data not shown).

## Discussion

We found that a lower CBF in patients with AD was associated with faster cognitive decline over a mean follow-up period of 2 years. This effect was strongest for lower parietal CBF and this association was independent of structural MRI measures for neurodegeneration and small vessel disease.

The major finding of this study is that CBF was independently associated with cognitive decline in patients with AD. This is a novel finding that seems in line with previous papers [15, 18] that report that a lower ASL-CBF predicts progression in cognitively healthy elderly and MCI patients. In addition, two studies using SPECT report a lower baseline CBF in faster declining AD patients compared with slowly declining patients [16, 17]. We found that a lower parietal CBF showed the strongest association with subsequent cognitive decline. A lower CBF in AD patients has been found to be most pronounced in posterior regions [7, 9, 31]. Moreover, the finding that lower CBF in these regions is associated with decline is in line with previous studies [16–18]. Several lines of research highlight the relevance of posterior brain regions in AD. EEG abnormalities have, for instance, been found to be most severe in the posterior regions [34] and atrophy in these regions has also been associated with more rapid disease progression in AD [35]. Overall, our findings indicate that posterior CBF can provide relevant information regarding disease progression in AD.

Contrary to our expectation, we found no cross-sectional association between a lower CBF and a lower score on the MMSE. Possibly the current patient selection (patients with available follow-up) may account for the discrepancy with our previous work [8, 31], as the selected patients had a slightly higher baseline MMSE. Moreover, we found no associations for PVC cortical CBF with cognitive decline and we feel currently not able to explain why the associations were different for uncorrected and PVC cortical CBF. Partial volume effects related to cerebral atrophy may hamper CBF measurement [32]. However, the association for whole brain and parietal CBF remained significant after adjustment for NGMV and MTA. Whereas this may seem contradictory at first sight, we also like to point out that among the many different methods that currently exist to apply PVC, there is still no perfect or gold standard [36]. By looking at uncorrected CBF we remain the closest to our original data and this seems, therefore, most useful for extrapolation to a clinical setting, as no additional processing is necessary.

Whereas absolute CBF values may vary across studies as a result of perfusion measurement techniques, a consistent finding is lower CBF values in AD patients compared to controls [7, 37]. Changes in CBF are generally tightly linked to changes in brain glucose metabolism, and a decrease in CBF is thought to reflect synaptic failure [10–12]. Synapse loss is assumed to be the most direct pathological substrate of cognitive decline in AD [38], and abnormalities in synaptic functioning are also assumed to cause network disturbance [39]. Connectivity research shows that highly active areas, among which the posterior regions, are in particular affected in AD [40]. The association that we found between decreased posterior CBF and cognitive decline may, therefore, reflect network disruption. Nevertheless, decreased CBF may also reflect the presence of vascular disease [9]. A considerable part of AD patients has concomitant cerebral amyloid angiopathy (CAA) [41]. Interestingly, cerebrovascular amyloid deposition is found to be largest in posterior brain regions. The importance of decreased posterior CBF for cognitive decline may, therefore, also be associated with the presence of CAA. Overall, we found, however, that the associations of a lower parietal and occipital CBF with cognitive decline were independent of MRI markers for neurodegeneration and small vessel disease.

A strength of our study is the availability of longitudinal cognitive data in a well-characterized set of AD patients. Another strength is the use of linear mixed models for the statistical analyses. These models allow patients to have variable numbers of follow-up assessment as they take into account that the estimate of cognitive decline is less precise when patients have fewer follow-up measurements. In addition, we used 3D pcASL with whole-brain coverage to study CBF. A major advantage for use in a memory clinic population is that ASL can be performed during the same scanning session as structural images.

A possible limitation is that we included a purely clinical sample. All included patients were asked to return to the outpatient clinic not solely for research purposes, but also as a part of the clinical routine. This might have induced a selection bias, as only patients for whom follow-up was thought to be relevant were invited for follow-up and could be included in the present study. Indeed we found that patients included in the present study had a slightly higher baseline MMSE, but we found no differences in any of the other characteristics. Since invitations for follow-up were made blinded to CBF values, this selection will not have confounded our results. A limitation with regard to the ASL is that, ideally multiple post-label delay times would be used to account for delayed transit times, as excessively long arrival times may result in regional underestimation of CBF. Nevertheless, the delay time of 2.0 s that we used is recommended for a memory clinic population and is assumed to limit the impact of variation in transit time on the measured CBF [25]. In addition, it is known that caffeine intake and medication use may influence CBF and hence ASL measurement. We did not correct for any of these effects. Moreover, using the MMSE as a measure for cognitive decline might be considered a limitation as well, as this is a rather crude measure of cognition. Nonetheless, the MMSE is a generally widely accepted test for the evaluation of cognition in elderly patients and is easy to obtain, thus maximizing the number of patients with available data.

Previous studies showed that CBF starts to decrease early in the process of AD and that CBF decreases precede structural brain volume changes [8, 42]. Our current results seemingly fit with the notion that decreasing CBF, similar to other measures of synaptic failure [13, 14] or network dysfunction [43], does not reach a plateau early in the disease, but is associated with ongoing cognitive decline once patients are diagnosed with dementia due to AD. ASL scans are relatively easy to obtain and can be acquired during the same scanning session as structural MRI images and they may, therefore, be a promising additional tool. Whereas the associations that we found between lower CBF and faster cognitive decline were found in group-level analyses, perfusion measured with ASL may have prognostic value in individual patients as well. We found that the use of classifiers that predict diagnoses based on single-subject ASL are promising (Collij LE, Heeman F, Kuijer JP et al: Application of machine learning to arterial spin labelling in mild cognitive impairment and Alzheimer's disease. Radiology 2016; accepted). Possibly we will be able to develop classifiers that use perfusion MRI to predict cognitive decline. Overall, our results indicate that pcASL-CFB may have additive value to the conventional structural MRI measures: AD patients with a lower posterior CBF at the time of diagnosis show a more rapid cognitive decline.

## Abbreviations and acronyms

MMSE: Mini-mental state examination
MTA: Medial temporal lobe atrophy
PCASL: Pseudo-continuous arterial spin-labelling
PRCPCC: Precuneus and posterior cingulate cortex
PVC: Partial volume corrected
WMH: White matter hyperintensities
